# Supplementary material for: The Effect of the COVID-19 Pandemic on the Social Inequalities of Health Care Use in Hungary: A Nationally Representative Cross-Sectional Study
Source: Int J Environ Res Public Health. 2022 Feb 16;19(4):2258. doi: 10.3390/ijerph19042258 (PMC8872504; doi:10.3390/ijerph19042258)
Supplement: Supplementary file 1 [file ijerph-19-02258-s001.zip › ijerph-1549195-supplementary.pdf]

Table S1 Stratum specific GP visit frequency in a year observed in prepandemic and pandemic periods

Characteristics		Prepandemic prevalence*	Pandemic prevalence*	OR (95%CI)**
**Age groups**	18-34 years	766 (70.9)	54 (33.3)	reference
	35-64 years	2010 (75.3)	326 (52.3)	**1.256 (1.093-1.444)**
	65+ years	1475 (91.2)	181 (83.4)	**4.761 (3.922-5.779)**
**Sex**	Female	2415 (82.8)	342 (57.7)	reference
	Male	1836 (74.9)	219 (53.5)	**0.695 (0.620-0.78)**
**COPD**	No	4039 (78.5)	533 (55.1)	reference
	Yes	212 (94.2)	28 (80.0)	**4.037 (2.549-6.393)**
**IHD**	No	3935 (78.1)	502 (53.7)	reference
	Yes	316 (95.5)	59 (88.1)	**5.641 (3.688-8.628)**
**Hypertension**	No	2455 (71.3)	285 (41.2)	reference
	Yes	1796 (93.3)	276 (88.7)	**6.428 (5.414-7.631)**
**Diabetes mellitus**	No	3736 (77.4)	421 (49.7)	reference
	Yes	515 (95.0)	140 (90.3)	**5.687 (4.140-7.812)**
**Cancer**	No	4143 (78.9)	544 (55.5)	reference
	Yes	108 (93.9)	17 (77.3)	**3.436 (1.895-6.230)**
**Region**	Central-Hungary	1206 (78.7)	150 (49.3)	reference
	Central-Transdanubia	482 (81.3)	87 (79.1)	**1.506 (1.215-1.868)**
	Northern-Great-Plain	667 (79.8)	84 (56.8)	1.143 (0.955-1.369)
	Northern-Hungary	532 (79.0)	64 (53.3)	1.073 (0.886-1.300)
	Southern-Great-Plain	522 (79.1)	58 (43.6)	0.966 (0.800-1.166)
	Southern-Transdanubia	397 (78.1)	50 (58.1)	1.079 (0.871-1.335)
	Western-Transdanubia	445 (78.8)	68 (67.3)	1.189 (0.966-1.465)
**Education level**	Primary	827 (80.8)	121 (73.3)	reference
	Vocational	1026 (79.5)	176 (57.0)	**0.768 (0.641-0.920)**
	High school	1456 (80.1)	200 (50.3)	**0.753 (0.635-0.893)**
	Tertiary	942 (76.2)	64 (49.2)	**0.710 (0.590-0.855)**
**Marital status**	Married	2472 (79.5)	295 (55.1)	reference
	Single	727 (70.9)	72 (38.5)	**0.612 (0.532-0.705)**
	Divorced	345 (81.2)	76 (51.0)	0.873 (0.715-1.066)
	Widowed	621 (90.8)	116 (92.1)	**3.204 (2.489-4.122)**
	Married missed	86 (69.9)	2 (40.0)	NC
**Etrhnicity**	non-Roma	4167 (79.4)	529 (56.5)	reference
	Roma	76 (73.1)	32 (49.2)	**0.563 (0.409-0.774)**
	Roma missed	8 (61.5)	0 (0.0)	NC

* number of cases (and proportion as %) of positive outcomes

** odds ratios with 95% confidence intervals from logistic regression models

NC- not computable

Table S2 Stratum specific specialist visit frequency in a year observed in prepandemic and pandemic periods

**Characteristics**		**Prepandemic prevalence***	**Pandemic prevalence***	**OR (95%CI)****
**Age groups**	18-34 years	585 (55.2)	35 (22.2)	reference
	35-64 years	1664 (62.7)	208 (33.5)	**1.288 (1.128-1.469)**
	65+ years	1177 (73.0)	135 (62.5)	**2.448 (2.104-2.849)**
**Sex**	Female	2001 (69.0)	232 (39.5)	reference
	Male	1425 (58.9)	146 (35.9)	**0.703 (0.635-0.778)**
**COPD**	No	3228 (63.3)	355 (37.0)	reference
	Yes	198 (88.4)	23 (65.7)	**4.017 (2.837-5.689)**
**IHD**	No	3131 (62.7)	329 (35.5)	reference
	Yes	295 (89.1)	49 (73.1)	**4.527 (3.383-6.060)**
**Hypertension**	No	1966 (57.8)	191 (27.9)	reference
	Yes	1460 (76.0)	187 (60.5)	**2.533 (2.263-2.836)**
**Diabetes mellitus**	No	2965 (62.0)	271 (32.3)	reference
	Yes	461 (85.2)	107 (69.5)	**3.298 (2.701-4.026)**
**Cancer**	No	3319 (63.7)	363 (37.3)	reference
	Yes	107 (94.7)	15 (68.2)	**6.372 (3.589-11.313)**
**Region**	Central-Hungary	1075 (70.6)	98 (32.2)	reference
	Central-Transdanubia	374 (63.4)	64 (58.2)	0.932 (0.778-1.116)
	Northern-Great-Plain	519 (62.6)	65 (44.5)	**0.833 (0.710-0.977)**
	Northern-Hungary	420 (63.3)	31 (27.0)	**0.769 (0.648-0.913)**
	Southern-Great-Plain	397 (60.4)	37 (27.8)	**0.680 (0.574-0.805)**
	Southern-Transdanubia	300 (59.8)	34 (39.5)	**0.733 (0.607-0.886)**
	Western-Transdanubia	341 (61.0)	49 (49.0)	**0.808 (0.674-0.970)**
**Education level**	Primary	614 (60.1)	84 (51.5)	reference
	Vocational	790 (61.6)	115 (37.3)	0.920 (0.790-1.071)
	High school	1177 (65.6)	145 (36.8)	1.062 (0.919-1.226)
	Tertiary	845 (69.0)	34 (26.4)	**1.288 (1.097-1.513)**
**Martal status**	Married	2054 (66.6)	203 (38.0)	reference
	Single	533 (52.5)	42 (23.0)	**0.556 (0.487-0.634)**
	Divorced	293 (69.3)	49 (33.3)	0.905 (0.755-1.084)
	Widowed	475 (69.5)	82 (65.6)	**1.338 (1.136-1.576)**
	Married missed	71 (60.7)	2 (40.0)	NC
**Etrhnicity**	non-Roma	3372 (64.7)	354 (38.1)	reference
	Roma	47 (46.1)	24 (37.5)	**0.484 (0.354-0.661)**
	Roma missed	7 (58.3)	0 (0.0)	NC

* number of cases (and proportion as %) of positive outcomes

** odds ratios with 95% confidence intervals from logistic regression models

NC- not computable

Table S3 Stratum specific hospital admission frequency in a year observed in prepandemic and pandemic periods

**Characteristics**		**Prepandemic prevalence***	**Pandemic prevalence***	**OR (95%CI)****
**Age groups**	18-34 years	84 (7.7)	10 (6.2)	reference
	35-64 years	296 (11.0)	24 (3.9)	**1.321 (1.039-1.679)**
	65+ years	348 (21.4)	34 (15.7)	**3.234 (2.548-4.105)**
**Sex**	Female	424 (14.4)	41 (6.9)	reference
	Male	304 (12.3)	27 (6.6)	**0.854 (0.735-0.993)**
**COPD**	No	662 (12.8)	60 (6.2)	reference
	Yes	66 (29.3)	8 (22.9)	**2.990 (2.259-3.957)**
**IHD**	No	602 (11.9)	53 (5.7)	reference
	Yes	126 (38.1)	15 (22.4)	**4.485 (3.597-5.594)**
**Hypertension**	No	340 (9.8)	29 (4.2)	reference
	Yes	388 (20.1)	39 (12.5)	**2.429 (2.090-2.822)**
**Diabetes mellitus**	No	600 (12.3)	37 (4.4)	reference
	Yes	128 (23.7)	31 (20.0)	**2.359 (1.941-2.867)**
**Cancer**	No	677 (12.8)	58 (5.9)	reference
	Yes	51 (44.3)	10 (45.5)	**6.045 (4.279-8.541)**
**Region**	Central-Hungary	196 (12.7)	13 (4.3)	reference
	Central-Transdanubia	85 (14.3)	12 (10.9)	1.255 (0.969-1.625)
	Northern-Great-Plain	115 (13.7)	7 (4.7)	1.108 (0.873-1.406)
	Northern-Hungary	99 (14.6)	13 (10.9)	**1.286 (1.005-1.645)**
	Southern-Great-Plain	99 (14.9)	8 (6.1)	1.218 (0.949-1.563)
	Southern-Transdanubia	69 (13.5)	8 (9.3)	1.160 (0.878-1.534)
	Western-Transdanubia	65 (11.5)	7 (6.9)	0.950 (0.715-1.262)
**Education level**	Primary	199 (19.4)	22 (13.3)	reference
	Vocational	182 (14)	24 (7.8)	**0.646 (0.526-0.795)**
	High school	207 (11.3)	17 (4.3)	**0.489 (0.400-0.598)**
	Tertiary	140 (11.2)	5 (3.8)	**0.517 (0.412-0.647)**
**Martal status**	Married	404 (12.9)	31 (5.8)	reference
	Single	87 (8.4)	9 (4.8)	**0.630 (0.500-0.794)**
	Divorced	71 (16.6)	9 (6.1)	1.197 (0.926-1.547)
	Widowed	150 (21.9)	19 (15.1)	**1.953 (1.604-2.378)**
	Married missed	16 (12.6)	0 (0.0)	NC
**Etrhnicity**	non-Roma	705 (13.3)	62 (6.6)	reference
	Roma	20 (19.2)	6 (9.2)	1.294 (0.846-1.979)
	Roma missed	3 (23.1)	0 (0.0)	NC

* number of cases (and proportion as %) of positive outcomes

** odds ratios with 95% confidence intervals from logistic regression models

NC- not computable

TableS4 Stratum specific CRPNR frequency in a year observed in prepandemic and pandemic periods

**Characteristics**		**Prepandemic prevalence***	**Pandemic prevalence***	**OR (95%CI)****
**Age groups**	18-34 years	49 (6.6)	3 (3.4)	reference
	35-64 years	105 (5.0)	15 (3.8)	0.760 (0.544-1.063)
	65+ years	91 (6.1)	18 (8.6)	1.023 (0.727-1.438)
**Sex**	Female	142 (5.8)	24 (5.7)	reference
	Male	103 (5.5)	12 (4.4)	0.918 (0.719-1.172)
**COPD**	No	220 (5.3)	32 (4.9)	reference
	Yes	25 (11.7)	4 (12.5)	**2.402 (1.598-3.612)**
**IHD**	No	209 (5.2)	26 (4.1)	reference
	Yes	36 (11.0)	10 (15.9)	**2.505 (1.793-3.501)**
**Hypertension**	No	131 (5.3)	14 (3.6)	reference
	Yes	114 (6.1)	22 (7.2)	1.260 (0.99-1.603)
**Diabetes mellitus**	No	196 (5.1)	15 (2.8)	reference
	Yes	49 (9.3)	21 (13.8)	**2.258 (1.701-2.998)**
**Cancer**	No	239 (5.7)	32 (4.8)	reference
	Yes	6 (5.5)	4 (21.1)	1.448 (0.750-2.792)
**Region**	Central-Hungary	67 (5.4)	10 (5.0)	reference
	Central-Transdanubia	21 (4.5)	1 (1.1)	0.734 (0.452-1.192)
	Northern-Great-Plain	52 (7.8)	13 (12.7)	**1.639 (1.164-2.309)**
	Northern-Hungary	43 (7.7)	2 (2.6)	1.347 (0.921-1.97)
	Southern-Great-Plain	36 (6.6)	2 (2.1)	1.117 (0.749-1.667)
	Southern-Transdanubia	19 (4.6)	4 (7.5)	0.922 (0.572-1.486)
	Western-Transdanubia	7 (1.6)	4 (5.6)	**0.387 (0.204-0.735)**
**Education level**	Primary	83 (9.5)	23 (16.4)	reference
	Vocational	49 (4.7)	8 (3.7)	**0.405 (0.290-0.565)**
	High school	81 (5.6)	5 (2.0)	**0.455 (0.338-0.612)**
	Tertiary	32 (3.3)	(0.0)	**0.266 (0.177-0.399)**
**Marital status**	Married	126 (5.0)	10 (2.7)	reference
	Single	44 (6.2)	4 (4.0)	1.287 (0.917-1.806)
	Divorced	25 (7.1)	9 (8.7)	**1.636 (1.108-2.415)**
	Widowed	44 (7.0)	13 (10.9)	**1.676 (1.217-2.308)**
	Married missed	6 (6.2)	0 (0.0)	NC
**Ethinicity**	non-Roma	230 (5.4)	28 (4.3)	reference
	Roma	14 (16.1)	8 (18.2)	**3.624 (2.254-5.828)**
	Roma missed	1 (16.7)	0 (0.0)	NC

** number of cases (and proportion as %) of positive outcomes*

*** odds ratios with 95% confidence intervals from logistic regression models*

NC- not computable

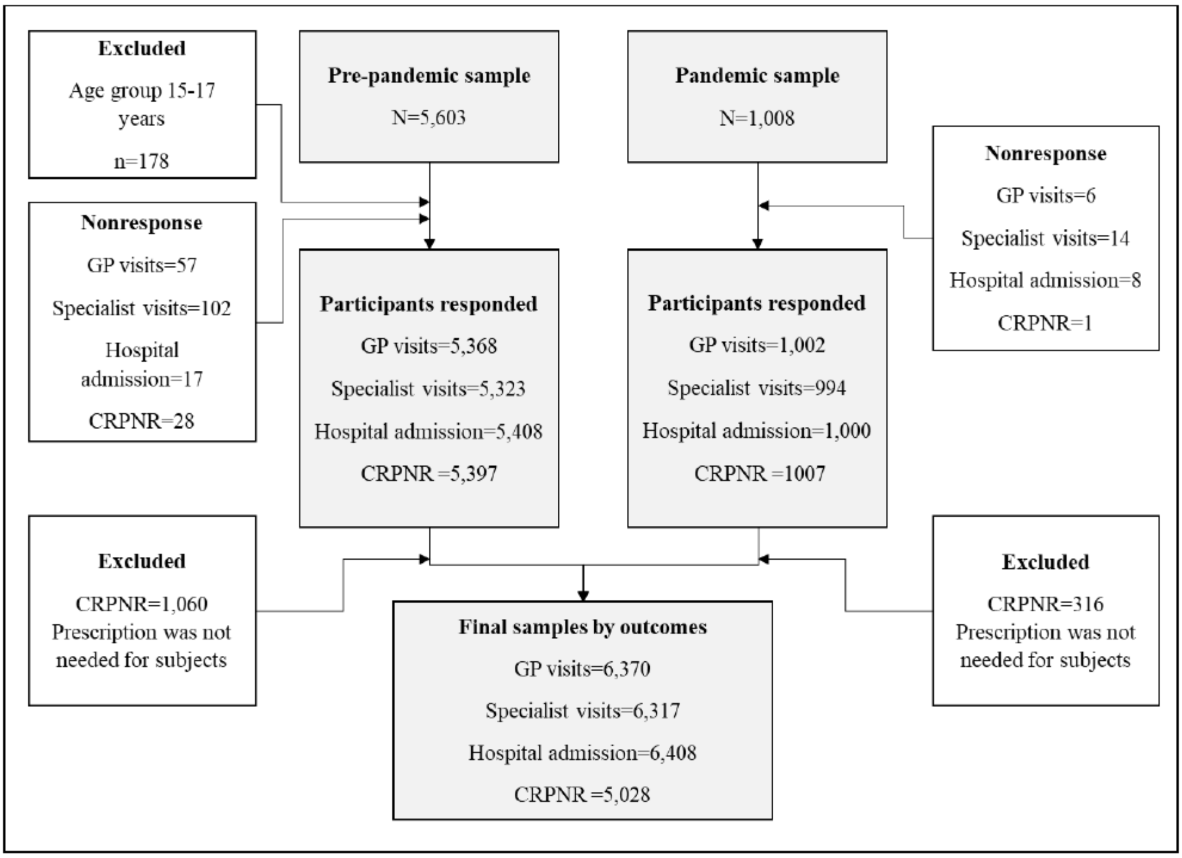

Figure S1. Sample recruitment flow and data cleaning techniques for each outcome variable (GP visits, specialists visit, hospital admission and CNRPNR) in pre-pandemic and pandemic periods.
